# Genetic Basis and Breeding Perspectives of Grain Iron and Zinc Enrichment in Cereals

**DOI:** 10.3389/fpls.2018.00937

**Published:** 2018-07-02

**Authors:** Ana Luisa Garcia-Oliveira, Subhash Chander, Rodomiro Ortiz, Abebe Menkir, Melaku Gedil

**Affiliations:** ^1^International Institute of Tropical Agriculture, Ibadan, Nigeria; ^2^Department of Genetics & Plant Breeding, Chaudhary Charan Singh Haryana Agricultural University, Hisar, India; ^3^Department of Plant Breeding, Swedish University of Agricultural Sciences, Alnarp, Sweden

**Keywords:** biofortification, cereals, iron, zinc, micronutrient deficiency, toxic risks

## Abstract

Micronutrient deficiency, also known as “hidden hunger,” is an increasingly serious global challenge to humankind. Among the mineral elements, Fe (Iron) and Zn (Zinc) have earned recognition as micronutrients of outstanding and diverse biological relevance, as well as of clinical importance to global public health. The inherently low Fe and Zn content and poor bioavailability in cereal grains seems to be at the root of these mineral nutrient deficiencies, especially in the developing world where cereal-based diets are the most important sources of calories. The emerging physiological and molecular understanding of the uptake of Fe and Zn and their translocation in cereal grains regrettably also indicates accumulation of other toxic metals, with chemically similar properties, together with these mineral elements. This review article emphasizes breeding to develop bioavailable Fe- and Zn-efficient cereal cultivars to overcome malnutrition while minimizing the risks of toxic metals. We attempt to critically examine the genetic diversity regarding these nutritionally important traits as well as the progress in terms of quantitative genetics. We sought to integrate findings from the rhizosphere with Fe and Zn accumulation in grain, and to discuss the promoters as well as the anti-nutritional factors affecting Fe and Zn bioavailability in humans while restricting the content of toxic metals.

## Introduction

From the 1950s onwards, the advancement in science and technology together with concerted efforts of international and national agricultural organizations has resulted in significant gains in world food production widely referred to as the “Green Revolution” (Ortiz, 2011). Globally, the availability of sufficient quantities of food is not only a simple achievement of the Green Revolution but has also helped to avert large-scale famines and social and economic upheavals (Khush, 1999). Without the Green Revolution, crop yields in Asia and Latin America would be at least 20% less, food prices would be up 19%, calorie consumption would be down by about 5%, and the number of malnourished children would be up by at least 2% (Evenson and Gollin, 2003). The widespread adoption of these technologies has made it possible to improve the *per capita* calorie consumption in different continents, especially in the developing world. Overall, the Green Revolution has paid rich dividends in food grain production, particularly cereals, that have led to a significant reduction in the proportion of undernourished people worldwide; however, the problem of malnutrition or lack of quality food still persists, leading to an economic burden for society (Pingali, 2012).

Anemia is the most common human nutritional malaise, resulting from iron (Fe) deficiency and affecting 32.9% people worldwide; meanwhile, zinc (Zn) deficiency affects 17% of the world's population, with the highest risk occurring in sub-Saharan Africa and South Asia (Wessells et al., 2012; Kassebaum et al., 2014). In the twenty-first century, there are strong concerns worldwide regarding the ability to produce nutritionally rich food because cereals are inheritably poor in essential micronutrients. Moreover, owing to a burgeoning human population and industrialization, this situation may be further compounded by the production of cereals in areas with low mineral phytoavailability (White and Broadley, 2009). Thus, there is an urgent global need to cope with the problem of micronutrient deficiencies that contribute to what is referred to as “hidden hunger” and affect at least 2 billion people (or 1 out of 3), mostly in sub-Saharan Africa, South Asia, and Latin America (FAO et al, 2015).

Diversity in diets in order to provide adequate micronutrient consumption is difficult to achieve in the developing world where resource-poor people cannot afford a variety of different foods. For example, many of the relatively cheap and staple crops such as cereals (Table 1), roots [cassava (*Manihot esculenta*z)], tubers [sweet potato (*Ipomoea batatas*) and yam (*Dioscorea* spp.)], and plantain (*Musa* spp.) that play a very important role in the daily diets of resource-poor people lack high enough amounts of micronutrients (Gibson et al., 2010). Hence, malnutrition and poor health affect these people, who may suffer from blindness or stunting, and sometimes even face death. To overcome this “hidden hunger,” medical supplements and fortification have been pursued (Underwood, 2000). In fact, food fortification has a long history of use in industrialized countries and relies on the addition of micronutrients to processed foods. However, food fortification tends to have a rapid but less sustainable impact, because various safety, technological, and cost considerations may place constraints on such interventions (Allen et al., 2006). Furthermore, such interventions do not always reach the desired target populations (Pfeiffer and McClafferty, 2007). By increasing the micronutrient content of energy-rich crops, micronutrient intakes among the poor can be increased, thereby leading to decreases in the prevalence of micronutrient deficiencies.

**Table 1:** Information and assumptions used to set target levels for mineral nutrient content in grains of biofortified staple cereals by CGIAR HarvestPlus.

**Crop**	**Average content at baseline (mg/kg)**		**Final target content (mg/kg)**		**Additional content through breeding (mg/kg)**	
	**Fe**	**Zn**	**Fe**	**Zn**	**Fe**	**Zn**
Rice	2	16	13	24	11	8
Wheat	30	25	52	33	22	8
Maize	30	25	52	33	22	8
Pearl millet	47	47	77	58	30	11
Sorghum	30	20	60	32	30	12

*Considering 90% retention of both Fe and Zn after processing, and 5 and 25% bioavailability for Fe and Zn, respectively, except Fe in rice grain where bioavailability is 10% (adapted from Bouis and Welch, 2010)*.

Biofortification is a strategy that involves the use of plant breeding or agronomic practices to increase the density of essential nutrients in the edible part of staple crops that may help to combat deficiencies among poor people who survive on main staples such as cereals (www.harvestplus.org). Agronomic biofortification is a fertilizer-based approach that relies on soil and/or foliar application of micronutrients either alone or in combination with other fertilizers. It is well-established that a Zn fertilizer strategy is an effective way to biofortify cereal crops with Zn, but recurrent cost is involved (Cakmak and Kutman, 2018). By contrast, genetic biofortification is a seed-based approach that complements agronomic biofortification and also current intervention methods such as supplementation and fortification of foods consumed daily. The aim of this strategy is to enhance the content and bioavailability of micronutrients such as minerals and vitamins in crops through plant breeding, thereby impacting favorably the diets of targeted populations, particularly the resource poor worldwide (Bouis and Welch, 2010). Because of its cost effectiveness, the 2008 Copenhagen Consensus ranked biofortification fifth for combating the world's greatest challenges (http://www.copenhagenconsensus.com/publication/second-copenhagen-consensus-biofortification-best-practice-meenakshi). Biofortifying staple crops through plant breeding is therefore a key option to improve micronutrient deficiency in human diets (Bouis and Saltzman, 2017).

## Relevance of Fe and Zn to human health

Among the mineral nutrients required by humans for their well-being, Fe and Zn play vital roles in numerous metabolic processes and are required in trace amounts by plants as well as animals (Welch and Graham, 2004). For instance, Fe is a well-known essential component of hemoglobin and myoglobin, which are involved in oxygen transport and storage. The greatest effects of Fe deficiency anemia are seen in females during adolescence and pregnancy. Likewise, Fe deficiency affects children's cognitive development until adolescence, and it increases their susceptibility to infectious diseases and mortality (Oliver and Gregory, 2015).

Similarly, Zn is also an essential cofactor for many enzymes and regulatory proteins, and it plays an important role in DNA as well as RNA synthesis and gene expression. Children show stunted growth and neurobehavioral difficulties resulting from Zn deficiency, which may increase the incidence and severity of diarrhea, among other conditions (Nriagu, 2007). Furthermore, Zn deficiency seems to be significantly related to anemia associated with Fe deficiency because Zn controls Fe absorption in the intestines (Chang et al., 2010; Graham et al., 2012). Hence, Fe and Zn are acknowledged as outstanding micronutrients owing to their importance in global public health.

## Physiology, genetics, and molecular aspects of Fe and Zn enrichment in cereal grains

For genetic biofortification, a better understanding of the key steps of mineral nutrient transport from the rhizosphere to grains is needed, which involves coordination of complex physiological steps such as acquisition of Fe and Zn in roots (uptake), subsequent long-distance transport from roots to shoots, and further redistribution toward the developing seeds (Zhao and McGrath, 2009; Carvalho and Vasconcelos, 2013). Although Fe and Zn are known to accumulate in grains, further insights regarding the underpinning physiology and genetics are yet to be revealed. Soil redox potential and pH affect uptake of Fe by roots and Zn accumulation in grains. Fe is mostly available in the rhizosphere as low solubility Fe^3+^ oxyhydrates, while Fe is oxidized in aerobic soils with high pH, thus occurring as insoluble ferric oxides. Free ferric Fe from the oxides become available at low pH for further uptake by roots (Lindsay and Schwab, 1982).

Plants have developed different strategies for Fe uptake from the rhizosphere: Strategy I involving ferrous Fe^2+^ (non-Poaceae) and Strategy II utilizing ferric Fe^3+^ (Poaceae), referred to as reducing and chelating strategies, respectively, or a combination of strategies I and II (Connorton et al., 2017). Poaceae family members such as rice (*Oryza sativa* L.), maize (*Zea mays* L.), and wheat (*Triticum aestivum* L.) follow Strategy II, and their root epidermis secretes phytosiderophores (PSs) that form stable Fe(III) chelates in the rhizosphere (Roberts et al., 2004). *TOM1* (Transporter of Mugineic acid family phytosiderophores)/*ZIFL4* belongs to the major facilitator superfamily (MFS) that exports Fe^3+^-PS chelates in rice and barley (Nozoye et al., 2011). From the rhizosphere, these Fe(III)-PS complexes can be taken up into root cells by Yellow Stripe-Like proteins (YSLs). The maize oligopeptide transporter *YS1* is the founding member of the YSL family, which facilitates Fe^3+^-PS complex uptake from the rhizosphere, and subsequently the role of *YSL15* has also been confirmed in rice (Curie et al., 2001; Inoue et al., 2009). Besides Strategy II, both rice and barley have a functional homolog of *IRT1* (*Iron-Regulated Transporter 1*) that allows direct uptake of Fe^2+^ from the rhizosphere, thus clearly showing the different uptake strategies for Fe^2+^ and Fe^3+^ in these cereal crops.

Soil pH significantly influences Zn acquisition and uptake from the rhizosphere by roots because Zn binds tightly to soil elements and plant cell wall parts under high pH. However, under anaerobic conditions in soils, additional factors such as soil redox potential, total sulfur content, and soluble bicarbonate also affect the availability of Zn (Impa and Johnson-Beebout, 2012). As is also the case for Fe(III), which exhibits even lower solubility, Zn solubilization in the rhizosphere is thought to occur via plant-mediated acidification and secretion of low molecular weight organic chelator (Sinclair and Krämer, 2012). Details regarding the role and contribution of Zn acquisition by the plant remain unknown. The uptake of Zn may occur as a divalent cation (Zn^2+^) or as a Zn-PS complex formed with PSs known as Fe^3+^-chelators, which are secreted by roots of the plant (von Wiren et al., 1996). ZIP-like transporters may take up Zn as noted in Strategy I plants (Ramesh et al., 2003). Thus, modification of rhizosphere chemistry through root architecture and by secretion of more root exudates that can alter soil pH could be the first promising target for improving Fe and Zn acquisition in cereal roots.

After roots acquire Fe and Zn, their translocation to the shoot and further movement to other vegetative organs depend of several steps, going through symplast, xylem, and phloem. Physiological studies have indicated that chelating molecules such as citrate, nicotianamine, and mugineic acid play a vital role in symplast heavy metal homoeostasis including Fe and Zn. Both minerals move through the xylem into the shoot, where Zn can move as free ions or in a complex with organic acids, while Fe is chelated to organic compounds of low molecular weight that are subsequently translocated by the xylem and phloem to other plant organs (Rellan-Alvarez et al., 2010; Lu et al., 2013). In plants, leaves are the most important sink tissue for these minerals where they are required in the plastids and mitochondria for numerous enzymes essential for photosynthesis and other cellular metabolic processes (Gupta et al., 2015). The *FDR3* gene has an important role in transporting Fe (Green and Rogers, 2004); while for Zn transport, heavy metal ATPase (HMA), a member of the P1B ATPase family, is a likely candidate for performing this task (Eren and Arguello, 2004: Hussain et al., 2004). At the molecular level, a plethora of genes associated with influx and efflux transporters has been discovered and extensively characterized in plants, including cereals, and which are involved in translocation of these minerals (Kobayashi and Nishizawa, 2012; Ricachenevsky et al., 2015; Vasconcelos et al., 2017). Moreover, transcription factors have also been identified that regulate the genes involved in the uptake of Fe and Zn and synthesis of PSs in cereals. Despite the tremendous improvement in understanding those components participating in translocation, it remains difficult to define precisely the contribution of each of the components in the metal movement flux for each translocation step.

Plants remobilize and move nutrients from vegetative source organs into seeds during the filling of grains (Waters and Sankaran, 2011). Hence, the amount of both Fe and Zn in the cereal grain is dependent on the former physiological processes: firstly, their acquisition from the soil by roots, and secondly, transportation to the shoots and further remobilization of stored minerals from leaves when they senesce at grain filling. Despite the large amount of these minerals in the vegetative tissues of cereals, their remobilization from leaves is an important process (from senescence to grain filling), contributing to accumulation in the seeds. Fe and Zn accumulate throughout cereal seeds, being primarily concentrated in the aleurone and embryo parts and to lesser extent in the endosperm, except in rice and barley where Zn appears to be less strictly confined to the aleurone than Fe (Persson et al., 2009). From a biofortification perspective, the heterogeneous distribution of these essential mineral elements in cereal grains further complicates the situation for their efficient loading into the core endosperm (Cakmak et al., 2010). Fe, Zn, copper (Cu), and manganese (Mn) are micronutrients that primarily accumulate in the seed aleurone layer, where phytic acid (the main form of Pi storage in seeds) is a strong chelator of metal cations, binding them to form phytate, a salt of inositol phosphate (Raboy, 2009). However, recent studies have shown that Fe is mainly associated with phytic acid, while Zn is bound to proteins, which clearly suggests that Fe and Zn have a different speciation in cereal grain tissues (Persson et al., 2009; Kutman et al., 2010). Future research, hence, is needed to elucidate the molecular aspects of bivalent metal speciation including Fe and Zn in different tissues of seeds for efficient Fe and Zn biofortification strategies in cereals (Persson et al., 2016).

## Breeding perspective of Fe and Zn biofortification in cereals

To address the widespread prevalence of micronutrient deficiency, especially pro-vitamin A, Fe, and Zn, the Consultative Group on International Agricultural Research (CGIAR; https://www.cgiar.org) HarvestPlus in collaboration with international and national research institutes emphasized biofortification of staple food crops as a cost-effective, easily applicable, and sustainable approach to benefit low-income households. This may complement other efforts aimed at reaching rural populations in developing countries (www.harvestplus.org). Considering the average content of Fe and Zn in cereal grains and their retention after processing, as well as addressing the issues related to the bioavailability of these mineral nutrients, HarvestPlus established target levels of these nutritionally important traits in cereal grains (Table 1). The initial screening of a large amount of crop germplasm suggested the existence of substantial genetic variation for these traits in cereal crops and their wild relatives. Besides the complex nature of these traits, the assays required to measure micronutrient content in plant samples are tedious and costly.

**Figure 1:**
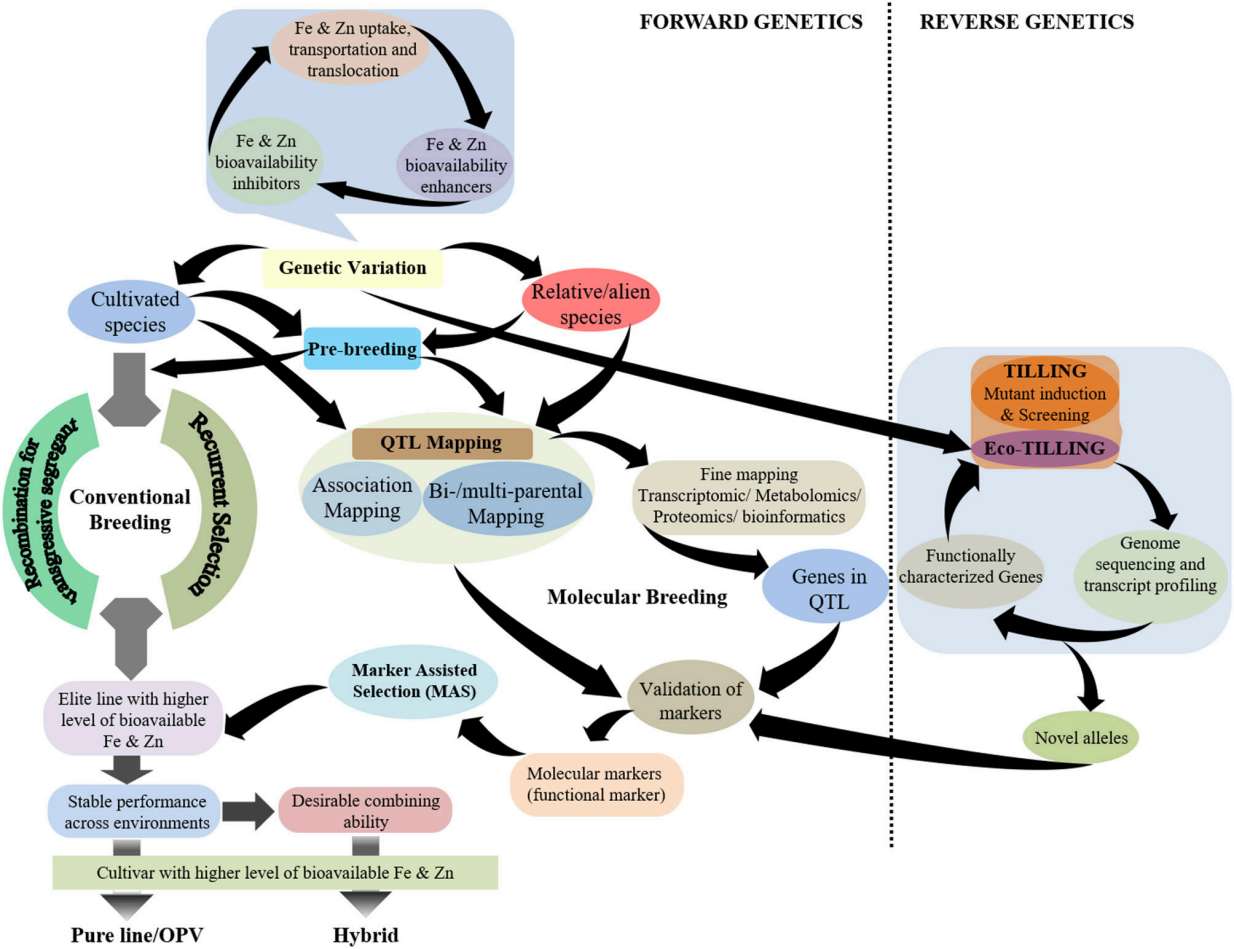
Schematic representation of breeding strategies using different genetic approaches for the development of grain Fe- and Zn-biofortified cereals.

While plant breeding has been pursued significantly to achieve biofortification in staple crops, the success in breeding for Fe and Zn biofortification in cereal crops lags behind the development of pro-vitamin A enriched cultivars of staple crops (Andersson et al., 2017). The possible reason for this is a better understanding of the carotenoid biosynthesis pathway in plants, especially in maize, that has led to the deployment of functional markers for pro-vitamin A biofortification in maize (Gebremeskel et al., 2018). Although, there have been significant advances in elucidating the mechanisms related to Fe and Zn homeostasis in model plants, detailed understanding is still lacking. It is noteworthy to highlight that several genes controlling Fe and Zn homeostasis in cereal grains—particularly rice—have been characterized, but their role in genotypic variation for the accumulation of these minerals in the grain remains unclear. Hence, a more holistic breeding approach is required for Fe and Zn biofortification of cereal grains that emphasizes the genetic enhancement of the contents of these minerals in cereal grains together with the factors that determine their bioavailability in humans such as inhibitors and/or enhancers (Figure 1). Further, there is the need to be cautious regarding inadvertent enhancement of non-essential/certain toxic elements, such as cadmium (Cd), in cereal grains.

## Exploring genetic variability for Fe and Zn enhancement in cereal grains

A pre-requisite for breeding for a specific trait is the availability of its genetic variation within the target gene pool. The task is somewhat complex while breeding for Fe and Zn biofortification in cereal grains because their concentration in the grain depends on various physiological processes. Plant breeders rely on additive genetic effects, transgressive segregation, and heterosis for improving desired traits when enough genetic variation exists. Recently, the genetic variability for these minerals in cereals, particularly maize, rice, wheat, barley (*Hordeum vulgare* L.), sorghum (*Sorghum bicolor* L.), and pearl millet (*Pennisetum glaucum* L.), which are the six most important crops and represent 89% of all cereal production worldwide, was reviewed, and the existence of significant genetic differences for these minerals was reported (Teklic et al., 2013; Goudia and Hash, 2015; Gregory et al., 2017). A survey of 1,400 improved maize genotypes and 400 landraces maintained at the genebank of the International Maize and Wheat Improvement Center (CIMMYT, El Batan, México) indicated about four- to six-fold variation for grain Fe and Zn (Bänziger and Long, 2000). Among the tropical-adapted maize inbreds of the International Institute of Tropical Agriculture (IITA, Ibadan, Nigeria), the best inbreds exhibited 32 to 78% more grain Fe and 14 to 180% more grain Zn over their trial mean (Menkir, 2008). Similarly, several-fold variation for grain Fe and Zn in disomic hexaploid bread wheat has also been reported (Velu et al., 2014; Goudia and Hash, 2015). Correspondingly, substantial variation for these minerals in rice grain has also been reported among different cultivars; however, grain polishing removed up to 50% of the Fe from the brown rice grain (Gregorio et al., 2000; Prom-u-thai et al., 2007). About, two-fold higher Zn concentration but slightly lower Fe concentration was reported in *indica* rice compared with *japonica* rice (Yang et al., 1998). Significantly lower Fe and Zn contents were found in the seed of modern cultivars of rice than in landraces (Anandan et al., 2011), thus arguing that breeders failed in introducing quality improvement, particularly for micronutrients, because they gave priority to other traits such as size, shape, and appearance of grain, milling quality, and cooking features. However, a notable aspect of the lower content of these nutrients in the seed of modern cultivars compared with landraces/germplasm may be the yield dilution effect; the total grain nutrient content may not differ significantly between landraces and modern cultivars, and part of this effect could be ascribed to higher grain yield in modern cultivars (Pfeiffer and McClafferty, 2007; McDonald et al., 2008). Therefore, grain yield must be kept in mind when discussing breeding solutions in cereals biofortification.

Simple and reliable phenotyping is always preferred by breeders, but an extensive survey of the literature pertaining to the existence of genetic variability for grain Fe and Zn contents in cereal crops clearly suggests that accurate measurement of these mineral nutrients is a challenging task. For the measurement of Fe and Zn content in plants and related material, a wide range of analytical methods is available ranging from semi-quantitative [Perl's Prussian blue and diphenyl thiocarbazone-based dithizone] to fully quantitative [atomic absorption spectrometry, inductively coupled plasma-optical emission spectrometry (ICP-OES), ICP-mass spectrometry, near-infrared reflectance spectrophotometry, X-ray fluorescence spectrometry, elemental distribution maps secondary ion mass spectrometry, synchrotron X-ray, fluorescence spectroscopy, micro-X-ray fluorescence spectroscopy, and Laser-induced breakdown spectroscopy], which can differ substantially with respect to the many attributes describing method performance (Ihnat, 2003; Pfeiffer and McClafferty, 2007). Therefore, the choice of analytical method would depend on the purpose and precision required in estimation. Alternatively, non-destructive quantitative techniques could be the choice method from a breeding perspective, because initial screening for grain Fe and Zn content together with other mineral elements in an appropriately large number of breeding lines can be obtained with minimal or no sample preparation, thereby enabling the discarding of progenies with the lowest content of these mineral elements. There are numerous possibilities for introducing variation in the results of different studies. Firstly, sensitivity of the method used for the quantification of Fe and Zn contents. Secondly, improper postharvest handling of the samples has also been observed to give erroneous results while estimating grain micronutrient concentrations. Furthermore, it is noteworthy that the variability among microenvironments for Fe and Zn may be significant, and most of the research presenting extremely high or low values of these mineral nutrients is based on single-year data; thus, the results are affected by a significant confounded influence of the sampling and environment. Additionally, extremely high trial mean values of these nutrients reported in some studies appear to be affected by the prior use of manure at some locations, because the level of these nutrients in cereal grains, particularly Zn content, can be even lower when grown in infertile/Zn-deficient soils (Cakmak et al., 2010; Xu et al., 2011; Velu et al., 2014).

## The value of wild relatives for Fe and Zn biofortification in cereals: opportunities for genetic gain

Modern cereal cultivars have a lower concentration of Fe and Zn in grains than landraces. This is because breeding has been mainly aimed at increasing grain yield or improving host plant resistance, among other target traits, instead of also improving the micronutrient concentration in grain. Utilization of landraces or crop wild relatives for genetic gain is not a new concept. The genetic variability for content of micronutrients is becoming acknowledged as a desired trait of crop wild relatives, particularly for rice and wheat.

The wild *Triticum* and *Aegilops* species have very high grain Fe and Zn contents when compared with both bread and durum wheat (Cakmak et al., 2000; Ortiz-Monasterio and Graham, 2000; Chhuneja et al., 2006; Rawat et al., 2009) as well as synthetic amphiploids (Calderini and Ortiz-Monasterio, 2003). Wild species such as *Triticum boeoticum, Triticum monococcum, Triticum dicoccoides* (wild emmer), *Aegilops tauschii*, and *Aegilops speltoides* were found to have substantially higher levels of these minerals (two- to three-fold) in their grain than modern wheat cultivars (Rawat et al., 2009; Xu et al., 2011; Velu et al., 2014). Compared with the alternative durum allele, recombinant chromosome substitution lines (RSLs) with *T. dicoccoides* carrying the *Gpc-B1* allele had a 12, 18, and 38% higher concentration of Zn, Fe, and protein content, respectively (Cakmak et al., 2004). High concentrations of Fe, Mn, and Zn in grain were stable across sites (Distelfeld et al., 2007). Hence, *T. dicoccoides* seems to be an interesting source for enhancing both protein and essential mineral content and concentration in wheat cultigens. Similarly, wild accessions of rice such as *Oryza rufipogon, Oryza nivara, Oryza latifolia*, and *Oryza officinalis* seem to be assets in rice improvement, showing higher values for Fe and Zn content than cross-bred cultivars (Banerjee et al., 2010; Anuradha et al., 2012).

## Identification of molecular markers for grain Fe and Zn biofortification in cereals

The finding of quantitative trait loci (QTLs) led to dissection of complex multigenic traits that were difficult to improve through crossbreeding before the progress made in DNA-aided analysis. QTL mapping for mineral nutrients in cereal grains has allowed the identification of many QTLs for both Fe and Zn (Table 2). Most of these QTLs, with a few exceptions, do not seem to be stable across sites. Furthermore, QTL mapping has also clearly indicated the role of epistasis in expression of these traits in cereal grains through interactions with other loci (Table 2).

**Table 2:** Main effect and epistatic quantitative trait loci (QTLs) associated with Fe and Zn accumulation in different tissues and their co-localization with other traits in cereal crops reported by different groups.

**Crop**	**Cross**	**Population type (size)**	**No. of MQTL for trait (PVE%, range)**	**No. of EQTL for trait (PVE%, range)**	**MQTL on chromosome (trait co-localizations)**	**References**
Rice	*indica* IR64 × *japonica* Azucena	DH (129)	3 GFe (14–18); 2 GZn (13–15); 2 GPhy (15–24)	–	12 (GFe, GZn)	Stangoulis et al., 2007
	*indica* Zhengshan 97 × *indica* Minghui 63	RIL (241)	2 GFe (11–26); 3 GZn (5–19)	7 GFe (5–12); 6 GZn (3–14)	–	Lu et al., 2008
	*indica* Teqing × *rufipogon*	IL (85)	2 GFe (5–7); 3 GZn (5–19)	–	9 (GFe, GCa); 12 (GZn, GCa, GP, GMg)	Garcia-Oliveira et al., 2009
	*indica* Bala × *japonica* Azucena	RIL (79)	4 GFe (10–21); 4 GZn (11–15); 3 LFe (10–12); 1 LZn (12)	1 GFe (–); 1 GZn (–); 1 LFe (–)	1 (GFe, GP, GCd, GPb, LPb, LP, LCu); 1 (LFe, LCd, GCu); 3(LFe, GCd, GMo); 4 (GFe, GPb); 6 (LFe, LMg, GZn); 7 (GFe, GZn, LZn); 10 (GZn, LSi)	Norton et al., 2010
	*japonica* JX17 × *indica* ZYQ8	DH (127)	2 GZn (11–12); 2 GCd/GZn (15–42)	–	–	Zhang et al., 2011
	*indica* Madhukar × *indica* Swarna	RIL (168)	7 GFe (7); 6 GZn (3)	–	7 and 12 (GFe, GZn)	Anuradha et al., 2012
	*indica* PAU201 × *indica* Palman 579	F_2_ (247)	8 GFe (2–27); 3 GZn (5–19)		10.1 (GFe, GZn, length/breadth ratio); 10.2 (GFe, GZn)	Kumar et al., 2014
Wheat	*Synthetic hexaploid wheat* W7984 × *T. aestivum* Opata 85	RIL (114)	1 ShFe (32.0); 2 ShZn (23–28)	–	–	Bálint et al., 2007
	*T. aestivum* Hanxuan10 × *T. aestivum* Lumai 14	DH (119)	4 GZn (5–12)	–	4A (GZn, GP)	Shi et al., 2008
	*T. boeoticum* Tb5088 × *T. monococcum* Tm14087	RIL (93)	3 GFe (7–13); 2 GZn (9–19)	–	7A.1 and 7A.2 (GFe, GZn)	Tiwari et al., 2009
	*T. aestivum* RAC875-2 × *T. aestivum* Cascade	DH (90)	1 GFe (–); 4 GZn (–)	–	3D (GFe, GZn)	Genc et al., 2009
	*T. durum* Langdon × *T. turgidum* ssp. *dicoccoides* G18-16	RIL (152)	11 GFe (2–18); 6 GZn (1–23)	–	2A.1 (GFe, GZn, GMg); 2A.2 (GFe, GZn, GPC, GS, GP); 2B & 7B (GFe, GMn); 3A (GFe, GMg); 5A (GFe, GZn, GPC, GCu); 6A (GFe, GPC, GCu); 6B (GFe, GZn, GCa, GCu); 7A (GFe, GZn)	Peleg et al., 2009
	*T. aestivum* Xiaoyan 54 × *T. aestivum* Jing 411	RIL (182)	2 GFe (3); 2 GZn (4–7)	1 GFe (11); 1 GZn (9)	4B, 5A, and 6A (GFe, GZn, GPC)	Xu et al., 2012
	*T. aestivum* Tabassi × *T. aestivum* Taifun	RIL (118)	6 GFe (9–47); 2 GZn (40–51)	–	–	Roshanzamir et al., 2013
	*Synthetic hexaploid* SHW-L1 × *T. aestivum* Chuanmai 32	RIL (171)	5 GFe (59–10); 4 GZn (6–9)	–	5B (GFe, GZn, GSe); 3D (GZn, GSe)	Pu et al., 2014
	*T. aestivum* Chuanmai 42 × *T. aestivum* Chuannong 16	RIL (127)	4 GFe (9–19); 3 GZn (14–16)	–	4D (GFe, GMn)	Pu et al., 2014
	*T. aestivum* PBW343 × *T. aestivum* Kenya Swara	RIL (177)	7 GZn (7–15)	–	2Bc (GZn, TKW)	Hao et al., 2014
	*T. spelta* PI348449 × *T. aestivum* HUW 234	RIL (185)	5 GFe (3–27); 5 GZn (5–16)	–	–	Srinivasa et al., 2014
	*T. aestivum* SeriM82 × *T. dicoccoides /Ae. tauschii* SHW CWI76364	RIL (140)	9 GFe (7–15); 4 GZn (8–20)	–	4BS (GFe, GZn, TKW); 6 BL (GFe, GZn)	Crespo-Herrera et al., 2016
	*T.aestivum* Adana99 × *T. sphaerococum* 70711	RIL (127)	7 GFe (9–17); 10 GZn (9–31)	–	2B and 6B (GFe, GZn)	Velu et al., 2017
	*T. durum* Saricanak98 × *T. dicoccon* MM 5/4	RIL (105)	4 GFe (10–17); 3 GZn (9–12); 2 Zneffi (6–9); 3 ShZn (8–15)	–	–	Velu et al., 2017
	*T. spelta* Bubo × resynthesized *hexaploid wheat* Turtur	RIL (188)	3 GFe (5–10); 4 GZn (3–17)	–	–	Crespo-Herrera et al., 2017
	*Synthetic hexaploid wheat* Louries × *T. spelta* Bateleur	RIL (188)	7 GFe (6–21); 12 GZn (3–33)	–	3B.1 and 3B.2 (GFe, GZn)	Crespo-Herrera et al., 2017
Barley	*H. vulgare* Clipper × *H. vulgare* Sahara 3771	DH (150)	5 GZn (14–27); 1 ShZn (50); 2 ClZn (15–30)	–	–	Lonergan et al. (2009)
	*H. vulgare* Scarlett × *H. spontaneum* ISR42-8′	ILs (54)	3 GFe (–); 3 GZn (–); 1 FlZn (–); 22 YlFe (–); 12 YlZn (–)	–	–	Reuscher et al., 2016
Maize	B84 × Os6-2	F_4_ (294)	1LFe (8); 3 GFe (7–8); 7 GFe/GP (7–11); 1 GZn (8); 1 GZn/GP (8)	–	5 (LCu, LFe, LMg); 2 (GFe, GFe/GP); 6 (GFe, GP, GFe/P); 4 (GZn, GP, GZn/GP, GMg/GP)	Šimić et al., 2011; Sorić et al., 2012
	Mu6 × SDM	F_2:3_ (189)	2 GFe (10–17); 3 CobFe (6–10); 4 GZn (10–13); 3 CobZn (8–15)	–	2 (GFe, CobFe, GZn, CobZn); 9 (CobFe, CobZn)	Qin et al., 2012
	Mo17 × SDM	F_2:3_ (189)	1 GFe (17–21); 4 CobFe (8–16); 3 GZn (6–21); 3 CobZn (7–20)	–	2 (GFe, CobFe); 10 (CobFe, CobZn); 9 (GZn, CobFe)	Qin et al., 2012
	178 × P53	F_2:3_ (218)	1 GFe (17); 4 GZn (6–18)	–	–	Jin et al., 2013
	B73 × Mo17 referred as IBM population	RIL (232–274)	5 GFe (9–12); 3 GbioFe (8–14); 3 GZn (5–10)	–	–	Lung'aho et al., 2011; Baxter et al., 2013
	IBM population	RIL (245)	2 LFe (2–10)	–	–	Zdunić et al., 2014
Pearl millet	ICMB 841-P3 × 863B-P2	RIL (144)	3 GFe (18–19); 2 GZn (20–50)	2 GFe (5–10); 7 GZn (6–10)	3 (GFe, GZn)	Kumar et al., 2016

*MQTL, main effect QTL; EQTL, epistatic QTL; GFe, grain iron; GZn, grain zinc; GPhy, grain phytic acid; LFe, leaf Fe; LZn, leaf Zn; Zneffi, Zn efficiency; ShZn, shoot Zn; ClZn, clum Zn; FlZn, flag leaf Zn; YlFe, young leaf Fe; YlZn, young leaf Zn; CobF, cob Fe; CobZn, cob Zn; GbioFe, grain bioavailable Fe; DH, double haploid; IL, inbred line; RIL, recombinant inbred line; F_n_, segregating offspring*.

Unfortunately, there is no literature indicating so far a success story for marker-aided selection (MAS) for improving Fe and Zn in cereal grains, but some progress has been made that has laid the foundation stone toward breeding for Fe and Zn biofortification in cereals using MAS. For instance, some of the QTLs identified for Fe and Zn are co-localized, thereby suggesting common mechanisms for their transport. Furthermore, some QTLs for these mineral nutrients are also co-localized with those for other mineral elements such as phosphorus (P) and calcium (Ca), or other agronomically important traits including grain protein content and grain weight (Table 2). Fine mapping of candidate genes related to various QTLs could be a further step for developing biofortified germplasm.

Rice is a model plant for cereal genetics. Chromosome 11 of rice bears a QTL for Zn concentration in the grain, which seems to be associated with *OsNAC5*—a transcription factor that appears to be related with the remobilization of Zn from green tissues to the seed (Lu et al., 2008; Sperotto et al., 2009, 2010). In unpolished rice grains, 10 candidate genes known for Fe and Zn homeostasis were localized in the QTL regions whereas another six candidate genes were close to QTLs on chromosomes 3, 5, and 7, respectively (Anuradha et al., 2012). Based on these results, Anuradha et al. (2012) emphasized the importance of candidate genes *OsYSL1* and *OsMTP1* for Fe; *OsARD2, OsIRT1, OsNAS1*, and *OsNAS2* for Zn; and *OsNAS3, OsNRAMP1, heavy metal ion transport*, and *APRT* for both Fe and Zn biofortification of grain in rice. Recently, Norton et al. (2014) also found several QTLs for grain Zn and other elements in diverse rice genotypes using genome-wide association mapping, but the known Zn-related genes were not found in these regions, thereby showing the novelty of their results.

The first QTL for grain Fe and Zn in wheat was found by Joppa et al. (1997), who mapped a major QTL (*Gpc-B1*) for grain protein content to chromosome 6BS in a population of recombinant inbred lines (RILs) that derived after crossing “Langdon” (LDN)—a durum wheat cultivar—and DIC6B—a chromosome substitution LDN line including wild emmer wheat. Subsequently, the *Gpc-B1* locus was also found to be related to high concentrations of both Fe and Zn, as well as with fast leaf senescence. The dissection of the *Gpc-B1* locus by positional cloning revealed that the gene underlying the *Gpc-B1* locus encodes *NAM1*, which is a NAC transcription factor that belongs to a protein group that includes “*No Apical Meristem*” (*NAM*) in *Arabidopsis thaliana* (Uauy et al., 2006; Distelfeld et al., 2007). The ancestral wild wheat allele *NAM-BI* leads to fast senescence and enhances the remobilization of nutrients from the leaves to the developing grains. Modern wheat cultivars have instead a non-functional *NAM-BI*. Both Fe and Zn can be manipulated together because of the co-localization of their QTLs (Shi et al., 2008), whose mapping was facilitated by using RILs or diverse double-haploid (DH) populations (Table 2). The identification and tagging of DNA markers related to both traits provides an aid for crossbreeding, thereby accelerating biofortification for Fe and Zn in grains of cereals.

## Breeding for enhancement of Fe and Zn bioavailability: role of inhibitors and promoters

The ultimate goal of the breeding for Fe and Zn biofortification in cereals is to satisfy the requirement of the human body for these minerals. Thus, the bioavailability of these minerals should be measured according to the cereal-based foods consumed rather than as their quantity in the cereal grains. Considering the low bioavailability of these minerals, it seems to be difficult to meet this demand alone by enhancing the grain Fe and Zn content in cereals. Hence, Fe and Zn should be easily absorbable in the intestines—a difficult task due to inhibitors (e.g., phytic acid) or promoters such as prebiotics enhancing their absorption in the gut—to ensure their effective availability from cereal-based diets (Roberfroid, 2007; White and Broadley, 2009; Dwivedi et al., 2014).

Phytic acid is an effective chelator of positively charged elements such as Ca, Fe, Mn, magnesium (Mg), potassium (K), and Zn, which after human or animal consumption binds to these minerals in the intestines forming mixed salts that are further excreted, thus resulting in mineral deficiency in human populations (Ali et al., 2010). Although phytate is considered an inhibitor of Fe and Zn bioavailability and therefore referred to as an anti-nutritional trait in cereal grains, it may have some health benefits such as being an antioxidant or anticarcinogen (Schlemmer et al., 2009). Furthermore, the important role of phytic acid has also been noted in plant traits such as seedling vigor or protection of seeds against oxidative stress during their lifespan (Doria et al., 2009). Hence, the existence of a minimum concentration of phytic acid in the cereal grains is still under scientific debate from health as well as crop performance perspectives.

Nonetheless, various low-phytic acid (*lpa*) mutants have been found in barley, maize, rice, and wheat exhibiting 50 to 95% reduced phytic acid P (Rasmussen and Hatzack, 1998; Raboy et al., 2000; Pilu et al., 2003; Shi et al., 2003; Guttieri et al., 2004; Liu et al., 2007). However, the pleiotropic effects of these *lpa* mutations resulted in significant grain yield loss and also affected other agronomic traits such as poor seed germination along with low grain weight and starch accumulation, and poor plumpness, among other characteristics (Raboy et al., 2000; Pilu et al., 2003; Guttieri et al., 2006; Zhao et al., 2008). Thus, seeking available variability will assist in finding new genetic mechanisms that reduce phytate and avoid any grain yield penalty in cereals. About two-fold variation for seed phytate concentration has been observed in wheat and rice (Liu et al., 2006; Stangoulis et al., 2007). Interestingly, two QTLs for seed phytate concentration have been identified so far in rice; one each on chromosomes 5 and 12 accounting for phenotypic variance of 24 and 15%, respectively (Stangoulis et al., 2007). Genetic markers nearby these QTLs should be used for testing their efficacy as aids for selecting low-phytate lines.

With the growing awareness about diet-related health problems, the presence of health-promoting natural compounds in staple foods, which was earlier considered of minor importance, has attracted greater attention in the food industry. Prebiotics are a group of carbohydrates that are known to confer benefits for human health by selectively promoting the growth or activity of gut microbiota (Dwivedi et al., 2014). Thus, prebiotics in cereal grains should be taken into account for enhancing Fe and Zn bioavailability while undertaking biofortification. To date, scarce research has reported the influence of prebiotics on the absorption of these mineral nutrients in humans and the prevalence of the natural variation and inheritance of these compounds in cereal grains. There is, however, significant genetic variability for inulin concentration in the grains of maize and rice, both of which have lower inulin concentration that those of rye and wheat (Genc et al., 2005; Huynh et al., 2008a). Similarly, substantial genetic variation has also been reported in grain fructan content ranging from 0.7 to 2.9, 3.6 to 6.4, and 0.9 to 4.2% of grain dry weight in the different genotypic lines and cultivars of wheat, rye, and barley, respectively (Boskov-Hansen et al., 2003; Huynh et al., 2008a; Nemeth et al., 2014). There is a relatively high level of low molecular weight soluble dietary fiber in wheat. It includes fructan, which was found in a double mutant sweet wheat (SW) line; however, seeds were severely shrunken and shriveled, and had reduced kernel weight (Shimbata et al., 2011). Nevertheless, the SW mutant can be utilized in breeding programs as a novel source to raise grain fructan levels.

Among cereal crops, genetic mapping studies have been mainly performed in wheat for concentrations of grain prebiotics such as fructan, inulin, and arabinoxylan (Huynh et al., 2008b; Falcon, 2011; Nguyen et al., 2011). A total of five, four, and two QTLs explaining 2–27, 3–19, and 15–20% of phenotypic variation were detected in wheat for grain fructan, inulin, and arabinoxylan concentrations, respectively. Some epistatic QTLs were additionally detected for grain fructan and arabinoxylan concentration, although, their contributions were limited (Huynh et al., 2008b; Nguyen et al., 2011). Despite this, two QTLs each for fructan (6D and 7A), inulin (2BL.2 and 5BS), and arabinoxylan content (2A.1 and 4D.1) were major QTLs (PVE > 10%), suggesting molecular breeding to improve prebiotics significantly in grains of wheat. Recently, Huynh et al. (2012) mapped the fructan biosynthetic pathway gene coding for the enzyme sucrose:sucrose-1-fructosyltransferase (1-SST), which corresponds to the position of a major QTL on wheat chromosome 7A that affects the accumulation of grain fructan (Huynh et al., 2008b). Thus, identification of candidate genes underlying these QTLs would provide a basis for functional analysis and for the development of DNA markers that may assist molecular breeding with the aim of increasing prebiotic concentrations in the grain.

## Breeding for harmony between quality and safety of cereal grain

Besides food quality, food safety is also a “hot” topic that encourages scientists to engage in research related to health risks after consuming non-essential metals such as Cd and lead (Pb), and/or metalloids (arsenic, As), which have no beneficial role in plants, animals, or humans (Khan et al., 2015). Among these non-essential heavy metals, Cd particularly is known as highly phytotoxic, having a very low toxicity threshold level, and as a carcinogen, which is a great threat to human health. Nearly 27% of dietary Cd exposure is contributed by grain or grain products (Guttieri et al., 2015). Similarly, arsenic is also carcinogenic and can pose a serious threat to human health even at low concentrations. Moreover, the presence of high concentrations of these non-essential elements in cereal straw is still menacing because cereal straw is mainly used as livestock feed and thus these toxic elements may enter into the human food chain via contaminated meat or milk.

Soil is a natural source of heavy metals, and their elevated concentration in soil can occur either naturally or through anthropogenic activities such as urban and industrial activities as well as from agricultural practices. These toxic metals contamination is a non-reversible accumulation process due to their long estimated half-life in soil. Thus, accumulation of toxic metals in cereal grains impacts significantly on nutritional quality and crop safety. Generally, metals commonly enter plants as divalent cations. It has been reported that increasing accumulation of Fe and Zn in seeds leads to a higher accumulation of Cd, which chemically resembles Fe and Zn. Thus, uptake of Cd in roots and then translocation to seeds appears to occur inside plants along nutrient translocation pathways (Krämer, 2009). The first overlapping QTLs for essential and non-essential metals were identified in the Zn/Cd hyperaccumulator *Arabidopsis halleri*, and the candidate gene underlying the major QTL was identified as *AbHMA4* (*Heavy Metal ATPase 4*) (Hanikenne et al., 2008). Subsequently, *HMA2* was determined to contribute to Cd and Zn translocation in rice (Clemens et al., 2013).

The concentrations of essential mineral nutrients and non-essential metals in grains appear to be independently regulated because some independent grain Cd accumulation loci have been reported in cereals, such as the *Cdu1* locus on 5BL in durum wheat (Knox et al., 2009) and one major QTL on 5AL in bread wheat (Guttieri et al., 2015). The identification of causal genes underlying these QTLs will provide more biological insights into Cd accumulation in cereal grains. Similarly, rice genotypes having dysfunctional *OsNRAMP5* (Ishikawa et al., 2012) showed a substantial decrease in Cd uptake by roots, as well as Cd content in the straw and grain, but without decreasing the uptake of Fe by the roots, shoots, and straw (Ishimaru et al., 2012; Sasaki et al., 2012). These results suggest that a low grain Cd cereal cultivar can be developed without reducing the concentration of essential mineral nutrients through marker-aided breeding (MAB). Recent research has emphasized the importance of wild relatives for breeding high grain Fe and Zn in cereals crops. Nonetheless, possible pleiotropic effects of the introgression of elevated mineral nutrients need to be investigated by ICP–MS, thereby facilitating joint selection.

## Outlook

Globally, the committed efforts by CGIAR HarvestPlus have led to the integration of essential micronutrients as a core activity in the breeding programs of almost all major cereal crops. Considering the complex genetic mechanism of Fe and Zn accumulation in cereal grains, eradication of these mineral nutrient deficiencies by increasing their levels in cereal grains through conventional breeding is simply too difficult. In the post-genomic and computational systems biology era, the combination of high-throughput genomics and robust statistical analysis, particularly QTL mapping studies, has helped to dissect the molecular basis of natural diversity for complex quantitative traits in a better way. Recent molecular mapping studies clearly indicate the co-localization of QTLs for Fe and Zn with those for other potentially toxic metals such as Cd, Pb, and As. Available knowledge can be used to design targeted crosses for MAB targeting cereal cultivars with high levels of Fe and Zn.

Undoubtedly, QTLs detected only for Fe or Zn have also revealed that plants may be able to differentiate between nutrients and chemically similar toxin ions. Although no information is available so far about the enhancement of toxic metals in cereal grains through cross breeding, there is fear of inadvertent breeding for these non-essential metals that are toxic to both plants and animals even in low concentrations. Moreover, modifications in the accumulation of these toxic elements that are of concern for food safety are rarely determined during research on mineral nutrients. Thus, utilization of natural genetic variation for these mineral nutrients through a molecular breeding approach seems to be more attractive in the future. Furthermore, existence of substantial genetic variability for Fe and Zn bioavailability inhibitors and promoters also offers good opportunities to increase the bioavailable forms of these mineral nutrients in cereal grains. Genes accounting for this variability have rarely, however, been found and, therefore, are not yet being used in breeding; however, this also seems to be a promising approach for the near future. Hence, Fe and Zn bioavailability from cereal grains may be improved through breeding by accumulating either anti-nutrient agents or prebiotics. Furthermore, both functional and genetic evidence along with genome sequencing will provide means for gaining more insights regarding the emerging biofortification genomics.

## Author contributions

AG-O conducted the literature survey and together with SC wrote the first draft. RO edited and together with AG-O, SC, MG, and AM improved the manuscript writing. All authors read and approved the final manuscript.

### Conflict of interest statement

The authors declare that the research was conducted in the absence of any commercial or financial relationships that could be construed as a potential conflict of interest.

## Abbreviations

Fe: Iron
Zn: Zinc
CGIAR: Consultative Group on International Agricultural Research
QTL: Quantitative Trait Locus
MAS: Marker-aided Selection
MAB: Marker-aided Breeding
ICP-OES: Inductively Coupled Plasma-Optical Emission Spectrometry.
